# An engineered opsin monomer scrambles phospholipids

**DOI:** 10.1038/s41598-017-16842-z

**Published:** 2017-12-01

**Authors:** Kalpana Pandey, Birgit Ploier, Michael A. Goren, Joshua Levitz, George Khelashvili, Anant K. Menon

**Affiliations:** 1 Department of Biochemistry, Weill Cornell Medical College, 1300 York Avenue, New York, NY 10065 USA; 2 Department of Physiology and Biophysics, Weill Cornell Medical College, 1300 York Avenue, New York, NY 10065 USA; 3 Institute for Computational Biomedicine, Weill Cornell Medical College, 1300 York Avenue, New York, NY 10065 USA

## Abstract

The G protein-coupled receptor opsin is a phospholipid scramblase that facilitates rapid transbilayer phospholipid exchange in liposomes. The mechanism by which opsin scrambles lipids is unknown. It has been proposed that lipid translocation may occur at protein-protein interfaces of opsin dimers. To test this possibility, we rationally engineered QUAD opsin by tryptophan substitution of four lipid-facing residues in transmembrane helix 4 (TM4) that is known to be important for dimerization. Atomistic molecular dynamics simulations of wild type and QUAD opsins combined with continuum modeling revealed that the tryptophan substitutions lower the energetically unfavorable residual hydrophobic mismatch between TM4 and the membrane, reducing the drive of QUAD opsin to dimerize. We purified thermostable wild type and QUAD opsins, with or without a SNAP tag for fluorescence labeling. Single molecule fluorescence measurements of purified SNAP-tagged constructs revealed that both proteins are monomers. Fluorescence-based activity assays indicated that QUAD opsin is a fully functional scramblase. However, unlike wild type opsin which dimerizes *en route* to insertion into phospholipid vesicles, QUAD opsin reconstitutes as a monomer. We conclude that an engineered opsin monomer can scramble phospholipids, and that the lipid-exposed face of TM4 is unlikely to contribute to transbilayer phospholipid exchange.

## Introduction

Phospholipids flip-flop rapidly across disc membranes of retinal rod photoreceptor cells in an ATP-independent manner^1,2^. Biochemical reconstitution studies revealed that this phenomenon is due to the phospholipid scramblase activity of the G protein-coupled receptor (GPCR) opsin^3–5^. Purified opsin promotes transbilayer lipid exchange at a rate >10,000 s^−1^ when reconstituted into phosphatidylcholine liposomes. Scrambling is a constitutive activity of opsin^4^ that has been suggested to be necessary for homeostasis of photoreceptor disc membranes^5^. While the molecular basis of opsin-mediated scrambling is not known, two distinct mechanistic models have emerged^4,5^ in which the structural features needed for scrambling are proposed to be located either at the protein-lipid interface, or within the protein-protein interface of opsin dimers^6^. Differentiation between these models has been challenging because opsin dimerizes *en route* to reconstitution into lipid vesicles in which its scramblase activity is measured^4^, necessitating strategies to disrupt dimerization. A recent report indicated that opsin dimerization can be prevented by peptides that mimic transmembrane helices^7^; a similar peptide-based strategy was also deployed to disrupt dimerization of β2-adrenegeric receptors^8^. However, such peptides are not useful in clarifying the role of dimers in lipid scrambling as opsin-peptide complexes mimic dimeric interfaces that could potentially provide a lipid translocation pathway.

To address the role of opsin dimerization in lipid scrambling, we initially tested the scramblase activity of previously reported opsin mutants bearing amino acid substitutions in transmembrane (TM) helices 1 and 5 that have been proposed to be important for dimerization of opsin as well other GPCRs^9–12^. To this end, we used a reconstitution-based approach that was designed to reveal not only the effect of a particular mutation on opsin’s scramblase activity, but also to indicate with considerable precision the oligomeric state of the protein as it inserts into preformed vesicles during detergent-mediated reconstitution^13,14^. Using this approach we found that certain rhodopsin point mutants in TM1 and TM5 that are associated with autosomal dominant retinitis pigmentosa, reconstitute into vesicles as monomers but retain wild-type (WT)-like scramblase activity^13^. These studies indicated that opsin dimerization is not required for lipid scrambling and suggested a novel disease mechanism based on dimerization deficiency^13^. However, questions remained about how point mutations in specific TM helices can globally affect opsin’s ability to dimerize, and which structural elements of opsin are necessary (or dispensable) for lipid scrambling. To address these points, we considered that it would be important to target specific segments of the protein by rational mutagenesis with the goal of altering oligomerization properties in a predictive manner and studying the scrambling properties of the resulting oligomerization-deficient constructs.

In the present study we targeted TM4, a helix implicated in dimer formation across many Class A GPCRs^9–12^ that had not been considered in our initial studies^13^. We rationally designed an opsin variant (QUAD opsin) in which four lipid-facing residues in TM4 were modified to tryptophan. Molecular dynamics simulations combined with continuum modeling of the energetics of protein-lipid interactions indicated that the effect of the tryptophan substitutions is to lower the energetically costly residual hydrophobic mismatch between TM4 and the membrane, and thus to reduce the drive of QUAD opsin to dimerize via the TM4 interface. Single molecule fluorescence microscopy experiments revealed that both WT and QUAD opsins are monomers when purified in dodecyl-β-d-maltoside after expression in HEK293 cells, and fluorescence-based activity assays indicated that QUAD opsin scrambles phospholipids similarly to WT opsin. However, unlike WT opsin which dimerizes prior to insertion into phospholipid vesicles^13^, QUAD opsin reconstitutes into vesicles as a monomer. We therefore conclude that an engineered opsin monomer can scramble phospholipids, i.e. a dimer interface is not required for scrambling, and that the lipid-exposed face of TM4 is unlikely to contribute to transbilayer phospholipid exchange.

## Results

### Design of an opsin variant with impaired dimerization

**Figure 1:**
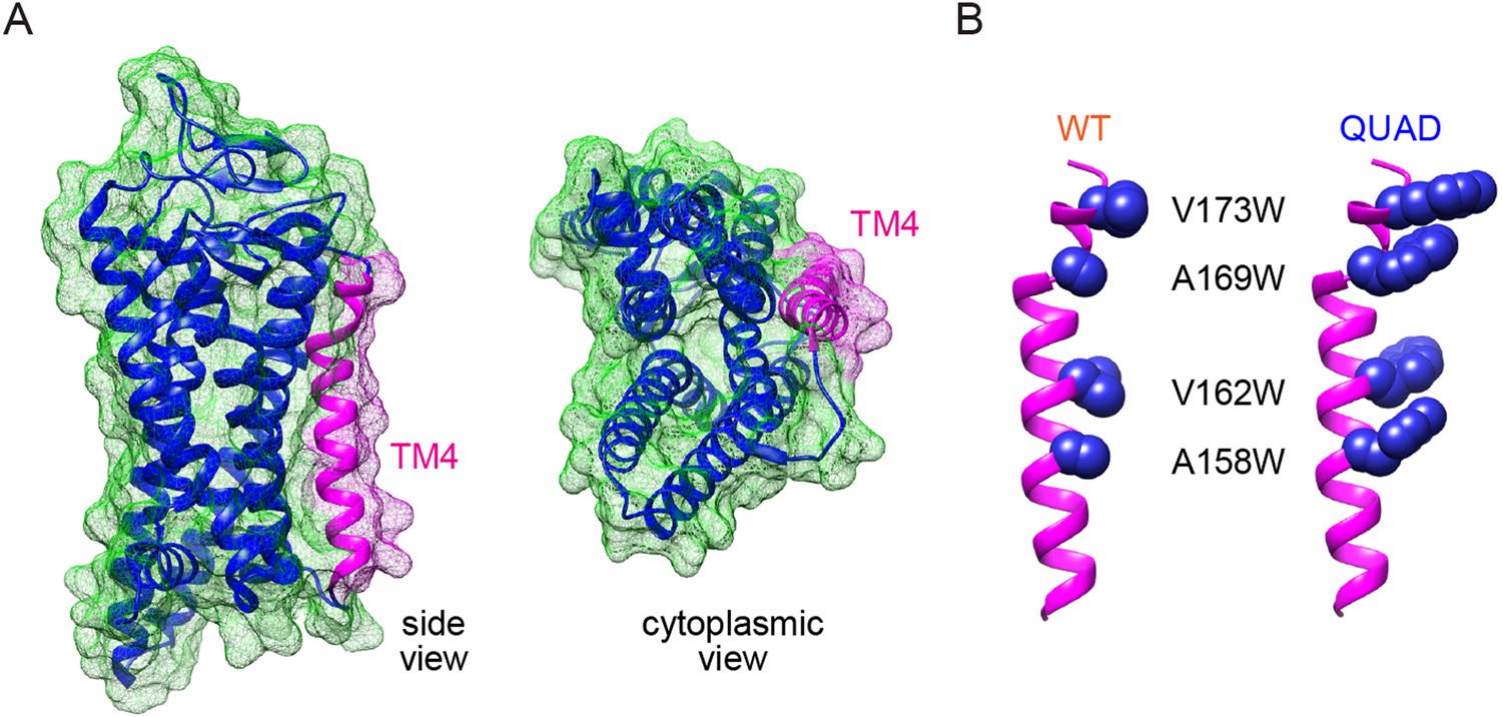
Comparison of molecular models of the TM4 helix in wild-type (WT) and QUAD opsin constructs. (**A**) Alternative views of WT opsin (PDBID: 4J4Q). The TM helices are shown as ribbons and green mesh represents the surface of the molecule. The TM4 helix is colored in magenta, while the rest of the protein is depicted in dark blue. (**B**) Views of the TM4 helix of WT and QUAD opsin highlighting (in blue space-fill) four residues, at positions 173^4.62^, 169^4.58^, 162^4.51^, 158^4.47^, that were substituted by tryptophan in the QUAD construct.

To design an opsin construct with impaired dimerization, we had the choice of modifying transmembrane (TM) helix 1 (TM1) and/or TM4, as both these helices have been strongly implicated in GPCR dimerization^9^. We targeted TM4 because it engages in fewer inter-TM contacts within the core helical bundle compared with other TM segments^15^ (Fig. 1A), suggesting that modifications to this helix would only minimally perturb the overall protein structure. Atomic force microscopy and electron cryomicroscopy studies identified TM4 residues 4.47, 4.51 and 4.58 as being important for rhodopsin dimerization^16^ (the residues are labeled according to the Ballesteros-Weinstein generic residue numbering scheme for GPCRs^17^), and crosslinking studies of the dopamine D2 receptor, another Class A (rhodopsin-like) GPCR, placed TM4 residues 4.51, 4.58 and 4.62 at the homodimer interface^18^. These residues correspond to amino acids on the outward-facing surface of TM4. We decided to substitute tryptophan (Trp) in place of these residues (4.47, 4.51, 4.58 and 4.62, corresponding to V173, A169, V162 and A158 in bovine opsin) (Fig. 1B). Because Trp has a bulky side-chain and is well tolerated in both hydrophobic and hydrophilic environments^19–22^, we reasoned that substitution of the selected residues by Trp would impact opsin dimerization without affecting the overall structure of the protein. Our expectation was that the quadruple mutant (QUAD opsin: V^4.62^W, A^4.58^W, V^4.51^W, A^4.47^W) would reconstitute into vesicles as a monomer, thereby providing us with the necessary tool to establish whether an opsin monomer can scramble lipids and potentially reveal whether TM4 plays a role in the scrambling process.

We first characterized QUAD opsin computationally by quantifying the energetics of protein-lipid interactions from analysis of atomistic molecular dynamics simulations combined with continuum mean-field modeling (CTMD)^23^. We analyzed how the Trp substitutions would affect residual hydrophobic mismatch (RHM), defined as the hydrophobic mismatch between a protein and lipid bilayer unalleviated by membrane deformations^24^. Minimization of the energetic cost related to RHM has been suggested to be a driving force underlying the association of multi-spanning proteins in lipid membranes^23–25^. Indeed, by identifying structural elements where the RHM energy penalty is largest, it is possible to predict specific modes of association^23^.

### Computational analyses suggest that QUAD opsin has a reduced tendency to dimerize

**Figure 2:**
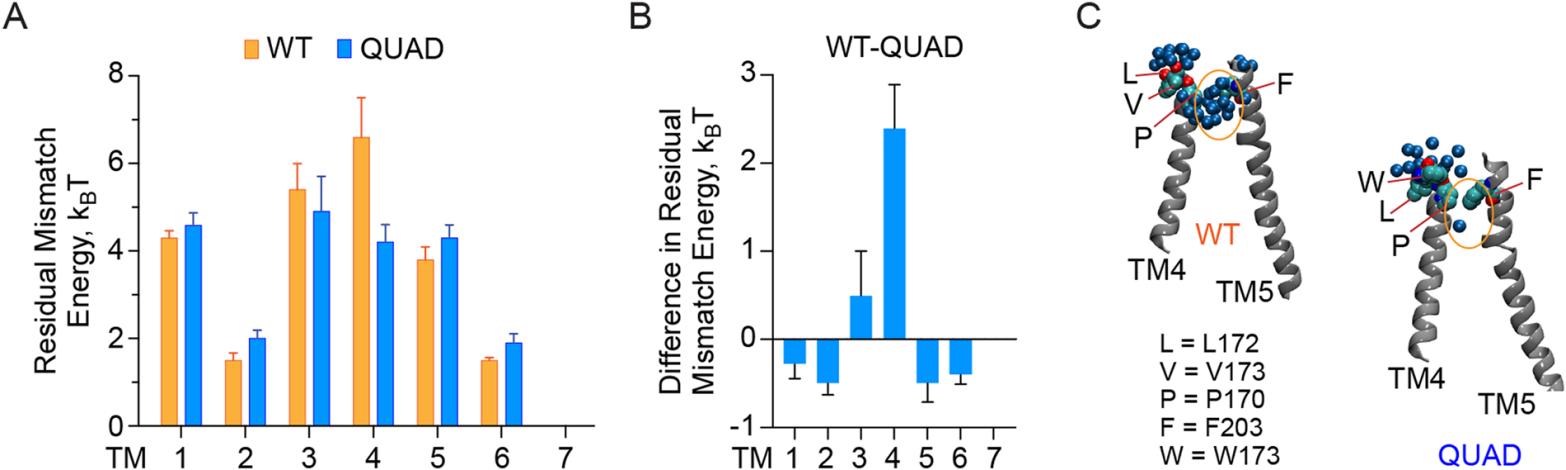
Residual hydrophobic mismatch (RHM) at TM helices in WT and QUAD opsin. (**A**) RHM energies were calculated at TM helices for WT and QUAD opsin, using microsecond-scale atomistic MD simulations (see Methods) in an explicit 9:1 mixture of 1-palmitoyl-2-oleoyl-sn-glycero-3-phosphocholine (POPC) and 1-palmitoyl-2-oleoyl-sn-glycero-3-(1′-glycerol) (POPG); error bars represent standard deviation of RHM measurements carried out on overlapping time intervals of the MD trajectory. (**B**) Difference in RHM energies between WT and QUAD opsin calculated from the data in panel A. The difference in RHM at the TM4 helix was statistically highly significant (p value < 0.002 from unpaired t-test). (**C**) Final snapshots from the simulations illustrating the source of RHM energies at TM4. Shown are TM4 and TM5 (in cartoon), and amino acid residues as indicated (van der Waals representation). Dark blue spheres are water oxygens within 5 Å of these residues. Water accumulation at the exoplasmic ends of TM4 and TM5 in WT opsin (region within orange oval) breaks hydrophobic contacts between P170 and F203, resulting in a large RHM at TM4. This hydrophobic contact is intact in the QUAD protein, thus reducing the RHM.

To quantify RHMs for WT and QUAD opsin, we first used the X-ray structure of WT opsin (PDBID: 4J4Q^26^) to build a 3D molecular model of QUAD opsin (see Methods). We then carried out extensive (totaling ~13 µs) atomistic molecular dynamics (MD) simulations of both WT and QUAD opsin in an explicit 9:1 (mol/mol) POPC/POPG lipid membrane and analyzed the resulting trajectories with a previously described CTMD protocol (see Experimental Procedures)^24^. The results show that TM4 has the largest RHM among all the TM helices in WT opsin (Fig. 2A), suggesting that TM4-mediated dimerization would provide a mode of association driven by RHM minimization. Furthermore, the RHM energy penalty is significantly lower at TM4 for QUAD compared to WT opsin (Fig. 2A,B). This is due to the different extent of water penetration between the extracellular ends of TM4 and TM5 (Fig. 2C) in the two constructs, resulting in larger RHM exposure at TM4 (at residue P170^4.59^ in particular) for WT opsin. RHM values for all other helices were similar (within k_B_T) for the two constructs (Fig. 2A,B). These results suggest that TM4 is an important driver of opsin dimerization in the membrane, and that the quadruple Trp substitutions in TM4 are expected to decrease the propensity for dimerization by lowering RHM. We therefore proceeded to test experimentally whether QUAD opsin (i) scrambles lipids and (ii) reconstitutes into vesicles as a monomer.

### Expression of QUAD opsin

**Figure 3:**
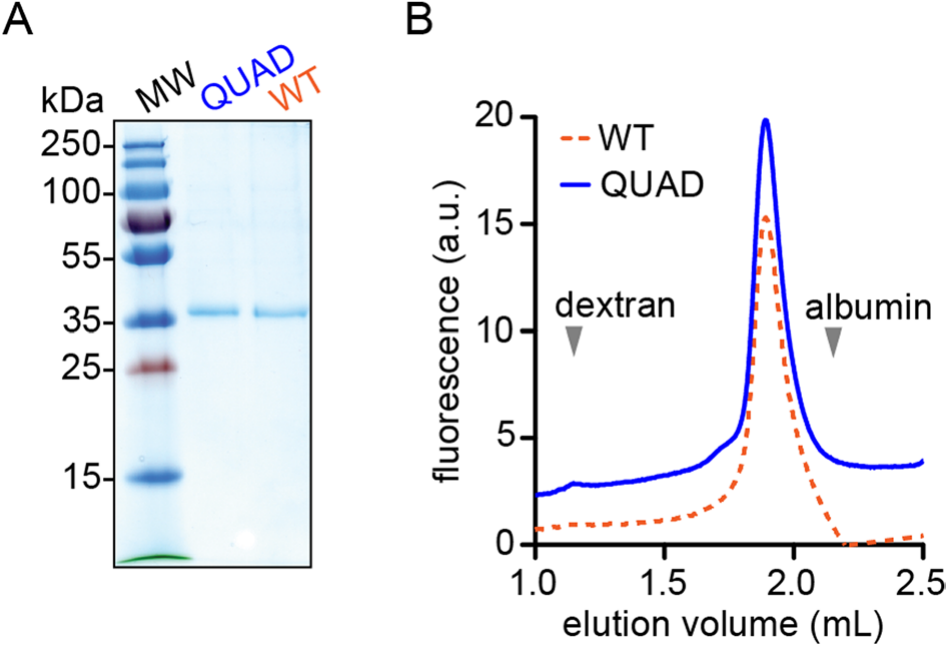
Expression of QUAD opsin. (**A**) Protein expression. WT and QUAD opsins were expressed as thermostable, FLAG-tagged variants in HEK293S GnTI^−^ cells, extracted with dodecyl-β-D-maltoside (DDM) and purified by FLAG affinity chromatography. An SDS-PAGE Coomassie-stained gel is shown of samples obtained from the same number of cell equivalents. (**B**) Fluorescence size exclusion chromatography (FSEC) analysis. GFP-tagged WT and QUAD opsins were expressed in HEK293S GnTI^−^ cells, and a DDM extract of the cells was analyzed (without purification of the expressed proteins) by FSEC. Both proteins display mono-disperse profiles (the trace for QUAD opsin is vertically displaced from that of WT opsin for clarity). Elution positions of dextran (void volume marker) and albumin are shown.

Using HEK293S GnTI^−^ cells for expression of homogeneously *N*-glycosylated proteins^27^, we obtained purified QUAD opsin in yields comparable to that of WT opsin (Fig. 3A). Analysis of GFP-tagged QUAD opsin by fluorescence size exclusion chromatography in 0.1% (w/v) dodecyl-β-d-maltoside (DDM) revealed a symmetric monodisperse profile identical to that of GFP-tagged WT opsin (Fig. 3B), indicating that the Trp substitutions in QUAD opsin do not affect the overall structure of the protein.

### Single molecule fluorescence experiments indicate that purified WT opsin and QUAD opsin are monomers

**Figure 4:**
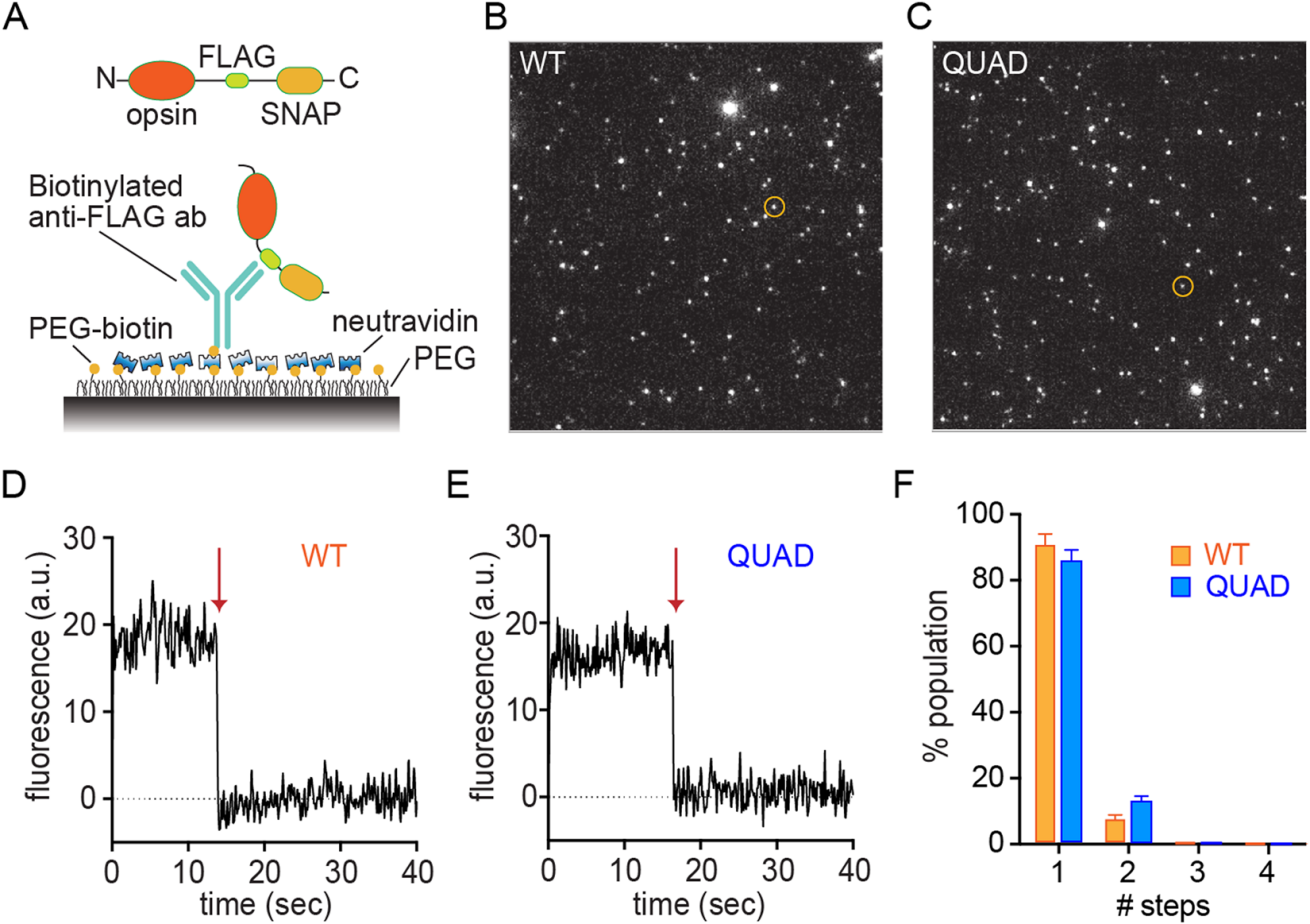
Single molecule fluorescence measurements reveal that purified WT and Quad opsins are monomers. (**A**) Schematic illustration of the single molecule pulldown (SiMPull) set up. (**B**) Representative TIRF image of SNAP-surface 549-labeled WT opsin-FLAG-SNAP; the circled spot corresponds to the photobleaching trace in panel D. (**C**) Representative TIRF image of SNAP-surface 549-labeled QUAD opsin-FLAG-SNAP; the circled spot corresponds to the photobleaching trace in panel E. (**D**) Trace depicting 1-step photobleaching of the circled spot from panel B when exposed to a 561-nm laser beam at time 0 s. The arrow depicts the point at which photobleaching occurred. (**E**) Trace depicting 1-step photobleaching of the circled spot from panel C when exposed to a 561-nm laser beam at time 0 s. The arrow depicts the point at which photobleaching occurred. (**F**) Fraction of the total population of spots that show 1-, 2-, 3-, or 4-step photobleaching (1102 and 1083 spots were analyzed for WT and QUAD opsin constructs, respectively). Error bars indicate standard errors calculated from 5 movies for each condition (169–257 spots were analyzed per movie).

It has been previously reported that opsin is monomeric when purified in 0.1% (w/v) DDM^4,13,28^ and we sought to confirm that this was also the case for our opsin variants. Single molecule subunit counting using photo-bleaching step analysis has been established as a useful approach to determine the distribution of oligomeric states of proteins^29,30^. Recently, single molecule pulldown (SiMPull) was introduced as a means of isolating individual protein complexes on a polyethylene glycol (PEG)-passivated coverslip at low densities for single molecule imaging analysis^31^. SiMPull has been successfully used in conjunction with subunit counting to analyze the monomer/dimer equilibrium of membrane proteins, including GPCRs^31,32^. In preliminary experiments, we analyzed GFP-tagged WT opsin expressed in HEK293T cells using SiMPull of fresh lysates in 0.1% (w/v) DDM and observed that 91% of the fluorescent spots were bleached in a single step indicating that the protein is a monomer (Supplementary Fig. S1). We next used this approach with affinity purified FLAG-SNAP-tagged WT and QUAD opsin (Fig. 4A) that we labeled with a benzylguanine-tagged red fluorophore (“SNAP-Surface-549”) via SNAP-tag chemistry^33^. The efficiency of SNAP-tag labeling under our reaction conditions is expected to be >80%^34^, comparable to the fraction of mature GFP in GFP-fusion proteins^29^. Individual proteins were immobilized using an anti-FLAG antibody (the antibody was biotinylated to enable its capture onto neutravidin-coated cover slips (Fig. 4A)) which interacts with the FLAG epitope situated between opsin and the SNAP tag in the fusion protein (Fig. 4A). Individual spots were visualized using total internal reflection (TIRF) microscopy (see Experimental Procedures; Fig. 4B,C). For most spots fluorophore bleaching occurred within 30 s (Fig. 4D,E) and bleaching analysis revealed that ~90% of spots bleached in 1-step (Fig. 4F), similar to our preliminary observation with WT-opsin-GFP (Supplementary Fig. S1). A small population of two-step bleaching spots was observed which is likely due to occasional coincidental localization of two proteins within a diffraction limited spot and/or because of the bivalency of the antibody. This background level of 2-step bleaching observed in our experiments (Fig. 4F) is consistent with previous studies with monomeric yellow fluorescent protein^31^. Our results strongly indicate that both purified WT and QUAD opsins are monomers when affinity purified in 0.1% (w/v) DDM.

### Scramblase activity of QUAD opsin

**Figure 5:**
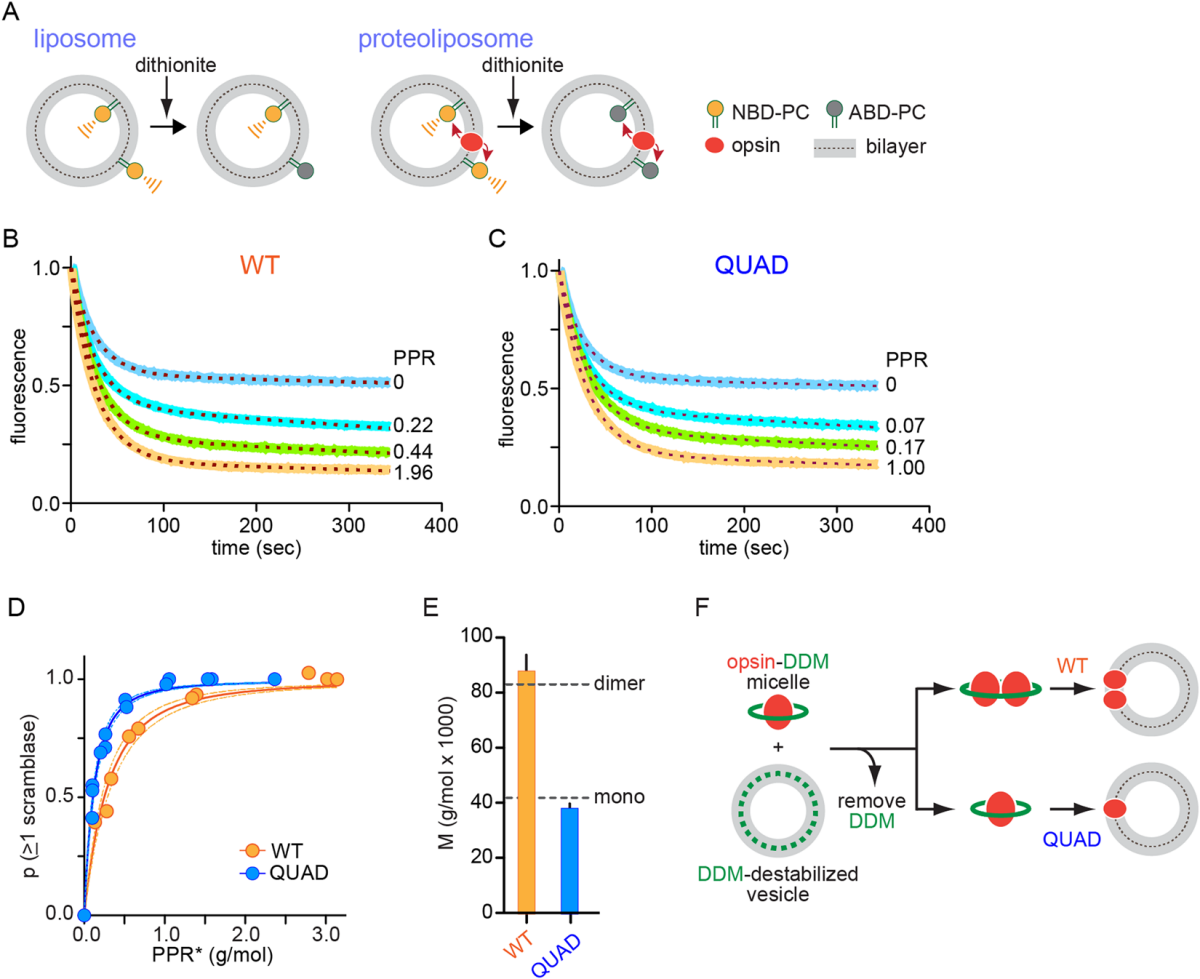
QUAD opsin scrambles lipids as a monomer. (**A**) Schematic representation of the fluorescence-based scramblase activity assay. (**B**) Representative fluorescence traces of scrambling by WT opsin, reconstituted at different protein/phospholipid ratios (PPR) into POPC:POPG (9:1) vesicles containing a trace amount of NBD-PC. (**C**) As in panel B, for QUAD opsin. (**D**) The extent of fluorescence reduction in the scramblase assay was determined for vesicles reconstituted with QUAD and WT opsin over a range of PPR values, 0–3 g/mol and the data were transformed into plots of p(≥1) scramblase (the probability of a vesicle having at least one scramblase) vs PPR* (related to measured PPR, see Experimental Procedures). The solid lines are data fits (Poisson analysis, see Experimental Procedures and Table 2), and the dashed lines are the 95% confidence interval for the fits. (**E**) Molar mass of the functionally reconstituted scramblase deduced from the data shown in panel D (see Table 2). (**F**) Schematic illustration showing that whereas both WT and QUAD opsin are monomers when added to DDM-destabilized phospholipid vesicles (based on Fig. 4), WT opsin dimerizes (multimerization is not shown here for simplicity) *en route* to reconstitution whereas QUAD opsin reconstitutes as a monomer (based on this figure, panel E). Direct evidence for dimerization of WT opsin during detergent withdrawal was previously obtained through co-immunoprecipitation studies^13^.

We next reconstituted QUAD opsin in large unilamellar vesicles to test its scramblase activity. For the scramblase assay (Fig. 5A), phospholipid vesicles are symmetrically reconstituted with a trace amount of fluorescent NBD-labeled phosphatidylcholine (NBD-PC) as well as the protein to be tested. NBD-PC in the outer leaflet of the vesicle is detected by dithionite, a membrane-impermeant reducing agent that eliminates NBD fluorescence. Treatment of protein-free vesicles with dithionite should lower fluorescence by 50% because NBD-PC molecules in the outer leaflet are reduced whereas those in the inner leaflet are protected. For vesicles reconstituted with a scramblase, e.g. WT opsin, dithionite treatment should result in 100% loss of fluorescence as NBD-PC can be exchanged between the inner and outer leaflet. In reality, the maximum extent of fluorescence loss does not exceed ~85% (Fig. 5B) because a fraction of vesicles is refractory to reconstitution^4,13,35^.

The extent of fluorescence loss in vesicles reconstituted with QUAD opsin was greater than that seen for protein-free vesicles (Fig. 5C) indicating that QUAD opsin - like WT opsin - is a scramblase. A control experiment using encapsulated NBD-Glucose^4,35^ confirmed that dithionite cannot cross the membrane of reconstituted vesicles (Supplementary Fig. S2), and that the only explanation for the greater extent of reduction of NBD-PC in WT-opsin and QUAD-opsin-containing vesicles is scrambling of the lipid reporter from the inner leaflet of the vesicles to the dithionite-accessible outer leaflet. The kinetics of NBD-PC fluorescence loss (t_1/2_ ~20 s) was the same for both opsin-containing vesicles and protein-free vesicles (Fig. 5B,C, Table 1) indicating that the rate at which dithionite reduces the NBD fluorophore is slow compared with the rate of scrambling. Within this limit of the time resolution of the assay we conclude that QUAD opsin and WT opsin have similar scramblase activity.

**Table 1:** Fit parameters for fluorescence reduction traces.

	n	Half-life (sec)	Magnitude of line slope S (sec^−1^ × 10^4^)
No protein	6	20.23 ± 1.79	1.20 ± 0.22
WT	20	22.63 ± 1.26	1.40 ± 0.16
QUAD	15	23.37 ± 0.87	1.40 ± 0.13

Fluorescence traces from scramblase assays performed over a range of PPR values, including those shown in Fig. 5, were fit to the equation F(t) = (1 − Plateau)^*^exp(−K^*^t) + Plateau − S^*^t, where F(t) = fluorescence at time t, t = 0 sec is the time of dithionite addition, and S = absolute value of the slope of the linear component. The results are provided as mean ± SEM (n = number of independent vesicle reconstitution samples). The standard error of individual fits was at least an order of magnitude lower than the SEM. Values of half-life (=0.69/K) and S are provided. The similar half-life values obtained irrespective of the PPR of the vesicles confirms that the dithionite reaction is rate-limiting in all cases. Only the plateau values (not shown here) vary with PPR – these are used to calculate the data fits described in Table 2.

### QUAD opsin reconstitutes into phospholipid vesicles as a monomer

We observed that we needed to reconstitute less QUAD opsin compared with WT opsin in order to obtain the maximum level of fluorescence reduction in the scramblase assay (Fig. 5B,C). As both proteins were reconstituted with similar efficiency (~70%, see Experimental Procedures) this observation suggested that on a per protein molecule basis, QUAD opsin could populate more vesicles and render them scramblase-competent than WT opsin. Because a single reconstitution event confers scramblase activity to a liposome^4^, the relationship between the fraction of scramblase-active liposomes and the protein to phospholipid ratio (PPR) of the reconstituted samples can be used to determine the molar mass of the functionally reconstituted protein. Using this approach, we showed previously that WT opsin reconstitutes into vesicles as a dimer, and to a small extent as a higher order oligomer^4,13^. We reinvestigated the reconstitution behavior of WT opsin in order to make a side-by-side comparison with QUAD opsin. Analysis of the data shown in Fig. 5D,E indicates that WT opsin functionally reconstitutes with molar mass 87.9 ± 5.79 kDa (±indicates standard error associated with the data fit), slightly higher than the mass of an opsin dimer (83.4 kDa), consistent with previous results (Table 2). In contrast (Fig. 5D,E), QUAD opsin functionally reconstitutes with molar mass 38.0 ± 1.73 kDa, equivalent to that of an opsin monomer (41.7 kDa) (Table 2).

**Table 2:** Analysis of p(≥1 scramblase) vs PPR* plots.

	*α* (x 104)	M (g/mol)	~n (Opsin n-mer)
WT	8.17 ± 0.54	87,905 ± 5, 810	≥2
QUAD	18.90 ± 0.86	38,000 ± 1, 730	1

The data were fit to equation 5 (Experimental Procedures) where α = 16/Mε^2^ is the fit constant, with units of mol.g^−1^.nm^−2^ and x is PPR*. See Experimental Procedures for details. M is the molar mass of the functional scramblase deduced from the fit, using ε = 0.472 nm as the cross-sectional radius of a phospholipid. The standard error associated with the fit is indicated. The molar mass of an opsin monomer is 41,700 g/mol.

## Discussion

Our data indicate that QUAD opsin scrambles phospholipids as a monomer (Fig. 5F) and that, within the limit of the time resolution of the scramblase assay, both WT and QUAD opsins are equivalently active in scrambling lipids (Table 1). Because the protein dependence plot in Fig. 5D has no inflection point we can rule out a scenario in which two or more QUAD opsins reconstitute independently into the same vesicle and subsequently dimerize in order to generate a functional scramblase; in this scenario, the protein-dependence plot would be sigmoidal, indicative of cooperativity, which is not the case here. Our observations clearly rule out a model of scrambling where lipid translocation necessarily occurs at the interface between protomers in an opsin dimer. This conclusion supports our recent report in which we showed that certain rhodopsin mutants associated with autosomal dominant retinitis pigmentosa also reconstitute into vesicles as monomers while retaining WT-like scramblase activity^13^. The combined results of the present and previous^13^ studies suggest that mutagenesis of any of the three TM helices implicated in dimerization (TM1 and TM5 (ref.^13^) and TM4 (present study)) globally affects the ability of opsin to dimerize via any of its potential dimerization interfaces as it transitions from a DDM-soluble monomer to a membrane-integrated protein during reconstitution (Fig. 5F). The mechanistic basis for this effect remains to be elucidated.

Importantly, while the bulky tryptophan substitutions of TM4 in QUAD opsin exert a profound effect on the magnitude of the residual hydrophobic mismatch between TM4 and the membrane (Fig. 2), thereby reducing the drive of QUAD opsin to dimerize via the TM4 interface and, indeed, affecting opsin dimerization via any interface (see above), the same substitutions have no detectable effect on the ability of the protein to scramble lipids. Thus, we suggest that an opsin monomer scrambles phospholipids without direct participation of TM4. Despite this advance, the molecular mechanism by which opsin translocates phospholipids remains elusive. Apart from several class-A GPCR proteins and the retinylidene protein bacteriorhodopsin that display opsin-like phospholipid scramblase activity when reconstituted into phospholipid vesicles^4,36^, the only other known phospholipid scramblases belong to the TMEM16 family of Ca^2+^-dependent ion channels and/or scramblases^35,37,38^. These proteins are homodimers, but each monomer possesses a membrane facing hydrophilic groove that likely provides the path for lipid headgroup and ion translocation^37–40^. Likewise, bacteriorhodopsin presents a series of membrane-exposed polar residues that could provide a transbilayer path for lipid translocation^36^. As opsin lacks these features, it may generate a translocation path dynamically, or scramble lipids by a mechanism that is distinct from that suggested for the TMEM16 scramblases and bacteriorhodopsin. For example, features of the opsin-lipid interface might create disturbances in the membrane that could promote scrambling. These disturbances could cause local thinning of the bilayer or create lipid packing defects. Interestingly, previous computational studies^41^ showed that rhodopsin is permissive to a surprisingly high degree of water permeation between its TM helices. These deeply penetrant water molecules may provide the route for lipid headgroups to negotiate the otherwise hydrophobic milieu of the interior of the membrane. Detailed evaluation of this concept by both computational and experimental approaches will be the subject of future work.

## Experimental Procedures

### Computational methods (protein constructs)

All computations were based on the X-ray structure of retinal-free opsin (PDBID 4J4Q). This structure also contains opsin-bound synthetic GαCT peptide^26^ which was not considered here. A quadruple mutant opsin (QUAD) with V173^4.62^W, A169^4.58^W, V162^4.51^W and A158^4.47^W mutations in TM4 helix (the numbers in superscript correspond to residue identity based on the Ballesteros-Weinstein generic residue numbering scheme for GPCRs^17^) was modeled using the homology modeling module of modeller software and the final model was chosen based on the best DOPE score^42,43^. The model for the QUAD mutant was energy minimized using TINKER molecular modeling software (http://dasher.wustl.edu/tinker/) and the OPLSAA force field^44^, before being used in molecular dynamics simulations (see below). RMSD of the QUAD model with respect to the WT structure, as calculated using Chimera software, was 0.07 Å for the backbone atoms.

### Computational methods (membrane-protein complexes)

Using CHARMM-GUI web facility^45–47^ the wild type (WT) and QUAD opsin molecular models were embedded into a lipid membrane consisting of a 9:1 mixture of POPC (1-palmitoyl-2-oleoyl-*sn*-glycero-3-phosphocholine) and POPG (1-palmitoyl-2-oleoyl-*sn*-glycero-3-phospho-(1′-*rac*-glycerol)) lipids. The protein to lipid ratio was 1:330. After adding a solvation box containing 100 mM NaCl the total system size was ~131,000 atoms.

### Molecular dynamics (MD) simulations

All-atom MD simulations of the WT and the QUAD constructs in the corresponding membrane environments were initiated with a previously established multi-step equilibration protocol^48^. During this stage, the backbone of the protein was first harmonically constrained and the constraints on the protein backbone were released gradually in three steps of 5 ns each, changing the restrain force constants from 1, to 0.5, and 0.1 kcal/ (mol Å^2^), respectively. This step was followed by unbiased MD simulations of the two proteins, ~250 ns for the WT protein and ~315 ns for QUAD opsin. The simulations were carried out using the NAMD 2.10 package and the latest CHARMM36 force field parameters for proteins, lipids, and ions^47,49^. The simulations implemented *rigidbonds* all option, 2fs integration time-step, PME for electrostatics interactions, and were carried out in NPT ensemble under semi-isotropic pressure coupling conditions, at a temperature of 298 K. The Nose-Hoover Langevin piston algorithm was used to control the target P = 1 atm pressure with the *LangevinPistonPeriod* set to 100 fs and *LangevinPistonDecay* set to 50 fs. The van der Waals interactions were calculated applying a cutoff distance of 12 Å and switching the potential from 10 Å.

After this equilibration phase, the velocities of all the atoms in the two systems (i.e. WT and QUAD opsin) were reset (at T = 298 K using random number seed) and 4 independent unbiased MD simulations per construct were carried out using ACEMD software^50^ resulting in a cumulative MD simulation time of ~13 µs (1.8 µs, 1.4 µs, 1.4 µs, and 1.5 µs for WT; 1.9 µs, 1.5 µs, 2.0 µs, and 1.4 µs for QUAD opsin). The simulations with ACEMD implemented CHARMM36 force fields, the PME method for electrostatic calculations, and were carried out according to the protocol developed at Acellera and implemented by us^50,51^ with 4 fs integration time-step and the standard mass repartitioning procedure for hydrogen atoms implemented in ACEMD. The computations were conducted under the NVT ensemble (at T = 298 K), using the Langevin Thermostat with Langevin Damping Factor set to 0.1.

### Energetics of membrane-protein interactions: calculation of Residual Hydrophobic Mismatch (RHM)

To quantify the energetics of hydrophobic mismatch between opsin and the lipid bilayer we used the Continuum-Molecular Dynamics (CTMD) approach described previously^24^. Briefly, CTMD calculates RHM, the hydrophobic mismatch unalleviated by membrane deformation. The RHM energies were computed as described^24,52–55^ from the surface area (*SA*_*res,i*_) of the *i* ^th^ residue participating in unfavorable RHM interactions. For hydrophobic residues, *SA*_*res,i*_ is the area of the residue that is exposed outside the hydrophobic core of the lipid bilayer. For polar residues, *SA*_*res,i*_ is the part of the residue that is exposed on the surface of the protein, but is situated within the hydrophobic core of the lipid bilayer. Practically, *SA*_*res,i*_ is quantified from residue-specific solvent accessible surface areas (SASA) obtained with NACCESS considering the solute as follows: i) the protein and the hydrophobic core of lipid bilayer (defined as the bilayer region within C2 lipid atoms), *SA*_*mem,i*_; ii) the protein only, *SA*_*prot,i*_. For hydrophobic residues

1 $$S{A}_{res,i}=S{A}_{mem,i}$$

and for polar residues

2 $$S{A}_{res,i}=S{A}_{prot,i}-S{A}_{mem,i}$$

The corresponding RHM energy penalty is directly proportional to *SA*_*res,i*_, and at a particular TM is given by

3 $${\sum }_{i=1}^{{N}_{res}}{\sigma }_{res}S{A}_{res,i}$$

where *N*_*res*_ is the number of residues in the TM and *σ*_*res*_ proportionality coefficient is taken to be 0.0028 kcal/(mol.Å^2^). As described previously^56^, interfacial Trp is not penalized as it can be favorably accommodated at the interface. RHM for Arg and Lys located close to the membrane headgroups is not considered as well since these amino acids alleviate hydrophobic mismatch by snorkeling^57^. Lastly, Ser and Thr are not penalized as their polar parts form H-bonds with the helix backbone of the protein^58^.

### Statistical analysis of the RHM results

RHM data for each protein construct were generated from the CTMD analyses performed on 4 independent MD simulation replicates (see above). Error bars reported in Fig. 2A,B are standard deviations from the mean calculated from a bootstrapping method involving calculating the mean from three randomly chosen replicates and repeating the procedure four times. Statistical significance of differences in RHM values at each TM helix between the WT and QUAD opsins was assessed by unpaired *t*-test.

### Vectors for opsin expression

We previously described a construct encoding C-terminal 3X FLAG-tagged thermostable opsin^4^. Modifications to this construct were accomplished by two-step overlap extension PCR and cloned into the NotI/EcoRI sites of the pMT3 vector. The sequence for each construct was verified at the Cornell University Life Sciences Core Laboratories Center. For GFP-tagged WT and QUAD opsins we used the pEGFP-N3 plasmid containing mouse opsin cDNA (kindly provided by Adam Smith (University of Akron))^59^. Mutations were introduced by a single two-step overlap extension PCR. PCR fragments were restricted with EcoRI/BamHI and inserted into pEGFP-N3. We previously described the SNAP-tagged wild type opsin construct^13^; the corresponding QUAD opsin construct was generated using the Gibson assembly cloning kit from New England Biolabs^60,61^.

### Opsin expression, purification and fluorescence labeling

Details of cell culture, protein expression, purification and quantification are provided elsewhere^4,13^. Briefly, WT and QUAD opsin were expressed as thermostable (N2C/D282C), C-terminally FLAG-tagged variants in HEK293S GnTI^−^ cells, purified by FLAG affinity chromatography^4^ and quantified by Coomassie-staining after SDS-PAGE, using an in-gel bovine serum albumin standard. The average yield for WT and QUAD opsin was ~6 µg per 10^7^ transfected cells. Thermostable, FLAG-tagged WT and QUAD opsins were also expressed with a C-terminal SNAP tag. These proteins were fluorescently labeled using SNAP-tag chemistry^33^; labeling was performed after capture of the protein on FLAG affinity resin, prior to elution of the protein with FLAG peptide. Briefly, after capture of the protein, the resin was washed with wash buffer (0.1% (w/v) DDM, 50 mM HEPES pH 7.4, 100 mM NaCl). SNAP-surface-549 dye diluted in wash buffer (~0.2 nmol per µg of protein) was then added and the sample was incubated overnight at 4 °C with end-over-end mixing. The resin was washed to remove unbound dye before eluting the labeled protein with FLAG peptide. Purified labeled protein was characterized by SDS-PAGE, using a Typhoon imager to visualize fluorescence and Coomassie staining to quantify protein as described above. SNAP labeling efficiency is expected to be >80% under our conditions^34^, comparable to the fraction of mature (fluorescent) GFP in preparations of WT-opsin-GFP^29^.

### Fluorescence size exclusion chromatography of GFP-tagged opsins

C-terminally GFP-tagged WT and QUAD opsins were expressed in HEK293S GnTI^−^ cells and extracted in buffer (50 mM HEPES, pH 7.4, 100 mM NaCl) containing 0.1% (w/v) DDM. After centrifugation to remove insoluble material, the supernatant was filtered and analyzed by fluorescence size exclusion chromatography on a Superdex 200 Increase 5/150 GL column (GE Healthcare Life Sciences) using a Shimadzu LC-20AD Prominence liquid chromatograph equipped with an RF-20A Prominence fluorescence detector (excitation λ = 395 nm, emission λ = 507 nm).

### Single molecule subunit counting

For subunit counting experiments, a passivated glass flow chamber was prepared as previously described^31^. Briefly, chambers were prepared using mPEG/biotin PEG-passivated quartz slides and coverslips. 0.2 mg/mL Neutravidin was added for 2 minutes at RT followed by washing with T50 buffer (10 mM Tris-HCL pH 8.0, 50 mM NaCl). To the Neutravidin coated chambers, 10–20 nM of biotinylated monoclonal anti-FLAG antibody (Sigma, cat. no. F9291) was added and incubated for 30 mins at RT followed by washing with T50 buffer. The flow chamber was placed on an inverted TIRF microscope (Olympus IX73 with cellTIRF system) and purified SNAP-surface549 (New England BioLabs) labeled QUAD-FLAG-SNAP or WT-FLAG-SNAP was immobilized at a density that allowed clear resolution of individual spots. The fluorophore was excited using a DPSS 561 nm laser, imaged through a 100x objective (NA = 1.49) and the data was collected at room temperature. Images were acquired with a scMOS detector camera (Hamamatsu ORCA-flash4.0 V3) at 20 Hz using Olympus cellSens software. Spots were analyzed using a previously described program^29^. Briefly, individual, immobile spots were identified and, after background subtraction and application of Gaussian weighting, manually characterized as bleaching in 1, 2, 3, 4, or 5 steps (or deemed uncountable). Data in bar graphs represent averages of the step distributions for different movies.

### Liposome and proteoliposome preparation

Unilamellar liposomes were prepared from a 9:1 (mol/mol) mixture of POPC and POPG (from Avanti Polar Lipids) as described^13,14^. The vesicles were reconstituted with opsin and C_6_NBD-PC (Avanti Polar Lipids) after detergent-mediated destabilization as described^4,13,14^. For experiments with NBD-Glucose (Invitrogen), NBD-PC was omitted. Recovery of protein and phospholipid after reconstitution was ~70%^13^.

### Scramblase assays and analysis of scramblase reconstitution

Scramblase activity was measured as previously reported, by adding dithionite to NBD-PC-containing vesicles and measuring the extent of fluorescence loss at the end point of the experiment (after >300 sec)^4,13^. Data were obtained for protein-free liposomes as well as proteoliposomes with a range of protein to phospholipid ratio (PPR, mg/mmol) values. The kinetics of fluorescence loss were evaluated as described in Table 1. End-point fluorescence reduction data were transformed as follows:

4 $${\rm{p}}(\ge 1\,{\rm{scramblase}})=({\rm{F}}\,-\,{{\rm{F}}}_{{\rm{o}}})/({{\rm{F}}}_{{\rm{\max }}}-\,{{\rm{F}}}_{o})$$

where F is the end point percentage fluorescence reduction, F_o_ is the percentage reduction obtained with liposomes (typically ~45%), F_max_ is the maximum percentage reduction observed for samples with high PPR (82.5%)^4,13^ and p(≥1 scramblase) is the probability that a particular vesicle in the ensemble contains at least one functional scramblase. The dependence of p(≥1 scramblase) on PPR follows Poisson statistics. Taking into account that a fraction of the vesicles is refractory to reconstitution (~35%), and that the vesicle population has a range of sizes described by a Gaussian distribution (${\rm{mean}}\,{\rm{radius}}\,\overline{r}=88\,{\rm{nm}}$ and standard deviation σ = 28 nm), p(≥1 scramblase) can be written as^13^:

5 $${p}(\ge 1\,{scramblase})=1-\frac{1}{\sqrt{1+784\alpha x}}\cdot {e}^{-3872\alpha x/(1+784\alpha x)}$$

where α is a fit constant that is inversely proportional to M, the molecular weight of the functional scramblase and x is PPR*, derived from the measured PPR after taking into account the fraction of vesicles that is refractory to reconstitution (PPR* = PPR/0.65). Fitting data sets of p(≥1 scramblase) versus PPR* yields α and therefore the molecular weight of the functionally reconstituted scramblase (Table 2)^13^.

### Data availability

The datasets generated during and/or analysed during the current study are available from the corresponding author on reasonable request.

## Electronic supplementary material

Supplementary Information
