# Supplementary material for: An engineered opsin monomer scrambles phospholipids
Source: Sci Rep. 2017 Dec 1;7:16741. doi: 10.1038/s41598-017-16842-z (PMC5711885; doi:10.1038/s41598-017-16842-z)
Supplement: Supplementary file 1 — Supplementary Information [file 41598_2017_16842_MOESM1_ESM.pdf]

Supplementary Information

An engineered opsin monomer scrambles phospholipids

Kalpana Pandey[‡1], Birgit Ploier[‡1], Michael A. Goren[‡1], Joshua Levitz[‡], George Khelashvili[†≠], and Anant K. Menon[‡*]

[‡] Department of Biochemistry, Weill Cornell Medical College, 1300 York Avenue, New York, NY 10065, USA
[†] Department of Physiology and Biophysics, Weill Cornell Medical College, 1300 York Avenue, New York, NY 10065, USA
[≠] Institute for Computational Biomedicine, Weill Cornell Medical College, 1300 York Avenue, New York, NY 10065, USA

* To whom correspondence should be addressed: Prof. Anant K. Menon, Department of Biochemistry, Weill Cornell Medical College, 1300 York Avenue, New York, NY, 10065, USA; Telephone +1-646-962-2476; E-mail: akm2003@med.cornell.edu

[1]These authors contributed equally to this work

Supplementary Figure S1

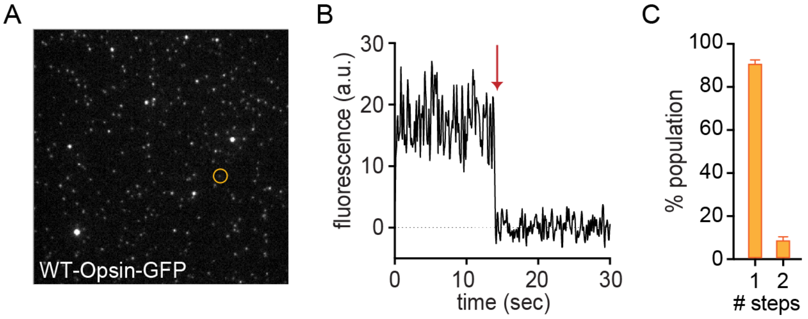

Supplementary Figure S1.
Single molecule photobleaching analysis of WT-Opsin-GFP.
WT-Opsin-GFP was expressed in HEK293T cells, extracted with 0.1% (w/v) DDM and tethered to a passivated glass slide using a biotinylated anti-GFP antibody. **(A).** Representative TIRF image of the sample showing distribution of spots. Panel B shows photobleaching of the spot circled in orange. **(B).** Representative trace showing single step photobleaching of a WT-Opsin-GFP molecule (corresponding to the circled spot in panel A). **(C)** Graph showing the fractional distribution of WT-Opsin-GFP molecules undergoing 1- and 2-step photobleaching in response to 488 nm illumination. 971 molecules were analyzed, 883 of which were bleached in a single step as shown in panel B**.** Error bars are standard errors calculated from 4 movies (>200 spots were analyzed per movie).

Supplementary Figure S2

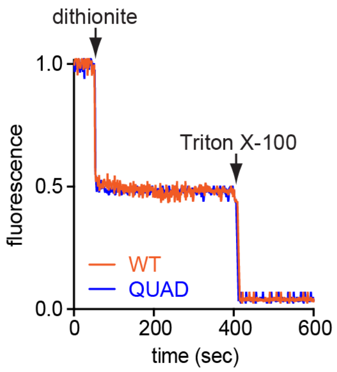

Supplementary Figure S2.
Reconstituted vesicles protect encapsulated NBD-glucose from dithionite.
2-NBD-Glucose (10 μM final concentration) and WT-opsin or QUAD opsin were added to DDM-destabilized vesicles and the samples were treated with BioBeads to reconstitute proteoliposomes containing encapsulated NBD-Glucose (PPR~1.1 mg/mmol (WT-opsin) and 0.45 mg/mmol (QUAD-opsin)). Fluorescence was recorded using the same settings as for NBD-PC (see Methods) and dithionite was added as indicated. For both WT-opsin and QUAD-opsin reconstituted samples, dithionite addition resulted in a sharp drop in fluorescence followed by a steady signal, indicating reduction of extravesicular NBD-Glucose and protection of the encapsulated pool. The extent of reduction was not as great as expected because of significant adsorption of NBD-Glucose to the BioBeads. Subsequent addition of Triton X-100 to disrupt the membrane barrier eliminated fluorescence. Thus, dithionite cannot cross the membrane of reconstituted vesicles to reduce trapped NBD-Glucose.
